# Meaningful Activities and Recovery (MA&R): the effect of a novel rehabilitation intervention among persons with psychiatric disabilities on activity engagement—study protocol for a randomized controlled trial

**DOI:** 10.1186/s13063-020-04722-3

**Published:** 2020-09-14

**Authors:** Siv Therese Bogevik Bjørkedal, Ulrika Bejerholm, Lene Falgaard Eplov, Tom Møller

**Affiliations:** 1 Team for Inclusion and Recovery, CORE: Copenhagen Research Center for Mental Health, Gentofte Hospitalsvej 15, opg. 3A, 2900 Hellerup, Denmark; 2 Department of Health Sciences, Mental Health, Activity and Participation (MAP), Centre for Evidence Based Psychosocial Interventions (CEPI), Lund University, Box 157, SE-221 00 Lund, Sweden; 3 CKO University Hospital of Copenhagen Rigshospitalet dep. 8513, 2100 Copenhagen East, Denmark

**Keywords:** Mental illness, Occupational therapy, Peer support, Rehabilitation, Recovery, Mental health, Wellbeing, Disability

## Abstract

**Background:**

Engagement in activities meaningful to the individual may support the process of recovery in those with mental illness. Persons with psychiatric disabilities may reduce their engagement in meaningful activities to various degrees with possible fluctuations over time. We hypothesized that activity engagement can be altered when opportunities and support are offered at an individual and a group peer-based level. Evidence is lacking regarding mental health interventions that enable engagement in meaningful activities, and powered effect studies are warranted.

**Methods:**

We propose an 8-month combined individual and group peer-based intervention, Meaningful Activities and Recovery (MA&R), and a study protocol for a multicentre two-armed parallel randomized controlled trial (RCT). The trial investigates the effects of MA&R in community mental health centres in Copenhagen and municipality services in Denmark. The trial will comprise 128 participants with psychiatric disabilities who will be randomized to one of two groups: (1) MA&R in addition to standard mental healthcare or (2) standard mental healthcare alone. The primary outcome is self-reported activity engagement, measured by Profiles of Engagement in People with Severe Mental Illness. Secondary outcomes are recovery, functioning and quality of life. Data will be collected at baseline and at follow-up at the end of the intervention.

**Discussion:**

This study adds new knowledge to a field with limited evidence, i.e. the clinical effectiveness of rehabilitation interventions among people with psychiatric disabilities, directly targeting activity engagement. The pragmatic design, regarding in- and exclusion criteria and settings, may allow assessment of the intervention’s effect under real-life conditions. The randomization, adequate power and fidelity monitoring allow testing of the intervention’s efficacy. The multicentre study design increases the potential for implementation in various mental health settings if the findings are positive. As the nature of the intervention does not permit blinding of the participants or staff, it may increase the risk of expectancy and performance bias. This must be considered when interpreting the findings.

**Trial registration:**

ClinicalTrials.gov NCT03963245. Registered on 29 May 2019

## Background

Psychiatric disabilities refer to inabilities and impairments when performing activities that the individual wishes or needs to perform in order to pursue life goals and fulfil social roles [1]. According to WHO’s International Classification of Functioning (ICF), performing activities is pivotal in enabling participation in major life areas, such as work, education, relationships, leisure and self-care [2]. ICF conceptualizes disability as a dynamic interaction between an individual’s health condition and contextual factors in that person’s life situation, e.g. coping style, social network and work. Substantial evidence shows that psychiatric disability is influenced by a complex interplay of symptoms, personal factors such as self-efficacy, and environmental conditions such as stigma or access to support [3–6]. Qualitative studies [7–14] in persons living with psychiatric disabilities have found that activity engagement is often reduced [7–9, 13, 14]. Simple activities, such as getting out of bed or leaving the house, can be arduous or sometimes impossible [8, 13, 14]. In some situations, inactivity may be therapeutic [8, 14]. In others, it may be detrimental, with isolation, preoccupation with thoughts, and lack of energy [9]. Inertia may be related to the illness leading to losses, for example, loss of identity, social status and close relationships; loss of future hopes and dreams; and loss of opportunities for work, education and material goods [8–10]. Living with mental illness can lead to isolation and fear. The risk of being overwhelmed, experiencing worsening of symptoms and facing stigma are aspects considered when engaging in daily activities [7, 9, 12, 15]. Thus, lack of activity engagement seems to be a key dysfunction related to psychiatric disability.

Observational studies suggest that the extent varies to which persons with psychiatric disabilities are engaged in activities. Higher levels of activities are associated with fewer mental health symptoms and better cognitive functioning, sense of coherence, self-mastery, empowerment and belief in one’s own work potential [16–19]. In RCT designs, one trial investigating the effectiveness of supported employment found that the intervention group increased their levels of activity engagement over 18 months [20]. In another, participants mapped their everyday activities, identified personal goals and planned strategies for change. They found this intervention more effective than care as usual regarding functioning and activity engagement [21]. However, there is currently a lack of evidence-based interventions that directly focus on activity engagement, and knowledge is lacking about how meaningful activity engagement can be supported as a crucial part of the recovery process for persons with mental illness [22–26]. This study builds on the evidence that occupational therapists (OTs) in mental health aim to enable participation in activities that are meaningful to the individual [27–29]. Moreover, we seek to imbed strategies of peer support since systematic reviews and meta-analysis suggest that peer support may foster hope, empowerment and social inclusion [30–33]. Hence, our study promotes peer support to enhance activity engagement by offering persons with psychiatric disabilities support to become active agents in their recovery process, to identify strengths and to break out of inertia and isolation [34]. The objective of the present study is to investigate an 8-month multifaceted intervention based on documented evidence from various psychiatric and occupational-based approaches. We aim to enable activity engagement in a diverse population of individuals with various psychiatric diagnoses and across healthcare and municipality settings.

### Objectives

This paper introduces a new rehabilitation intervention named Meaningful Activities and Recovery (MA&R) and presents a study protocol for a multicentre, two-arm parallel RCT to compare the effect of two interventions: (1) MA&R in addition to standard mental healthcare and (2) standard mental healthcare. This study protocol is reported according to the Standard Protocol Items: Recommendations for Interventional Trials (SPIRIT) 2013 statement [35, 36], and the study design is guided by the Consolidated Standards of Reporting Trials (CONSORT) criteria [37] for randomized trials of non-pharmacological treatment.

The primary objective of this RCT is to determine whether MA&R in addition to standard mental healthcare is more effective than standard mental healthcare alone regarding activity engagement (POES-S) [38, 39] in a sample of individuals living with psychiatric disabilities. The intervention is being tested in addition to standard mental healthcare as it is an add-on to usual care.

The second objective of this study is to evaluate whether MA&R in addition to standard mental healthcare is more effective than standard mental healthcare alone regarding outcomes related to activity engagement, e.g. functioning, personal recovery and quality of life [40–42].

## Methods

### Settings and participants

The study is conducted in Copenhagen Research Centre for Mental Health, CORE. The MA&R intervention is delivered in the five different sites where eligible participants will be recruited. The largest sites are two community mental health centres (CMHCs), situated in the Copenhagen Mental Health Center. The CMHCs offer outpatient services to persons with mental illness who live in central Copenhagen. The three other sites are municipality services in Copenhagen, Odense and Svendborg. The municipality services include rehabilitation services and drop-in centres for persons with mental health issues, and/or psychiatric disabilities, and are selected due to their contact with the study’s target group.

**Fig. 1:**
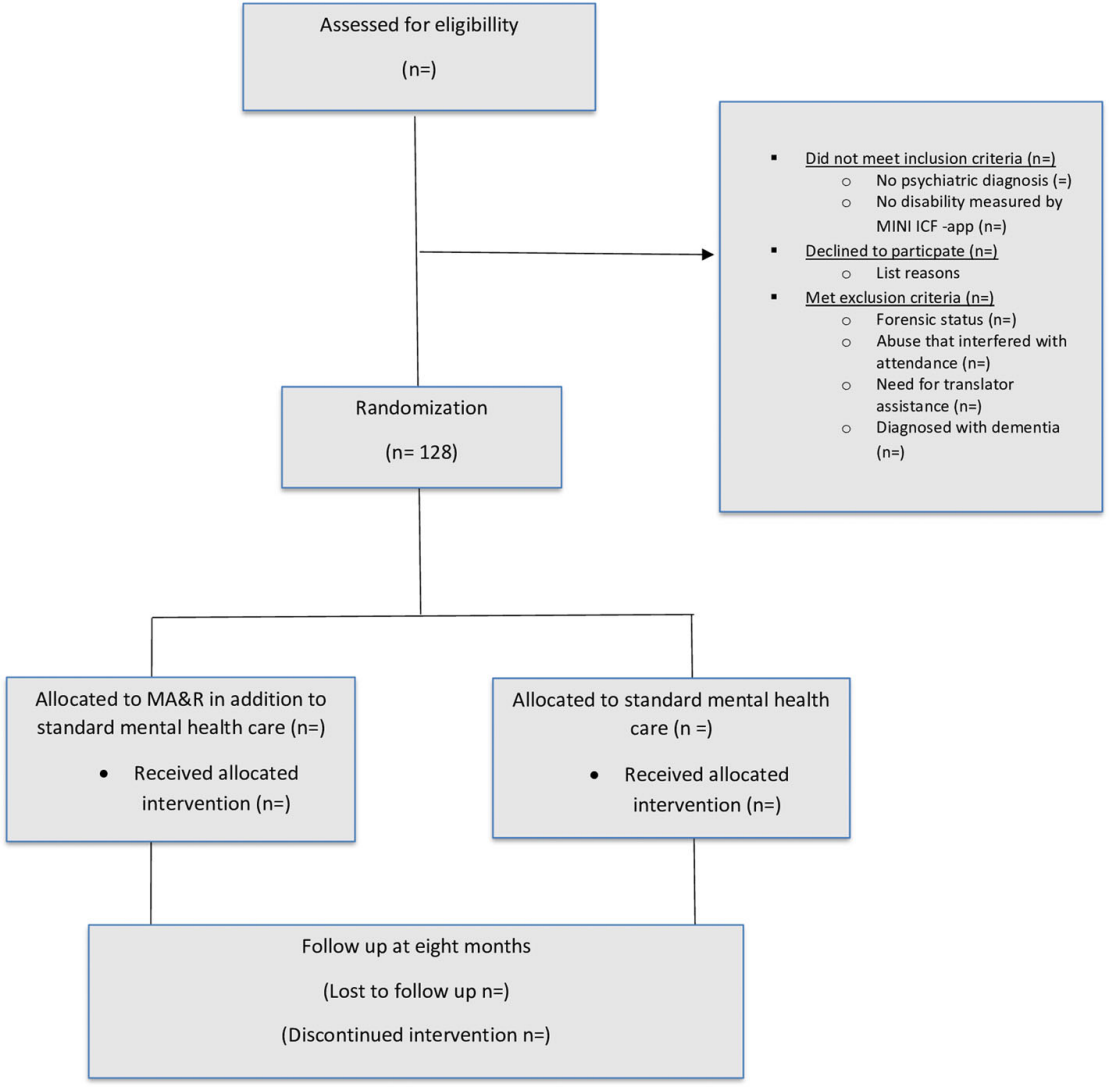
Displays the flow of participants from recruitment to study end

Eligible participants are identified by any professional at the respective sites or by self-referral (Fig. 1). The participant must be aged 18 years or older, have a psychiatric disability assessed by a researcher using the Mini-ICF Rating for Limitations of Activities and Participation in Psychological Disorders (Mini- ICF-APP) [43], be diagnosed with a mental disorder and provide informed consent. Persons ineligible for study participation include persons diagnosed with dementia, those with alcohol or substance abuse as a primary diagnosis or abuse that inhibits attendance at MA&R sessions, those with a forensic psychiatric status, those who require translator assistance (unable to speak Danish) or those who refuse to sign informed consent.

### Trial management

The principal investigator (LFE), co-investigators (TM and UB) and researcher (SB) communicate regularly through telephone conversations, e-mails or in person about preparation, revision and agreement of final protocol, publication of study papers and reviewing the progress of the study. The principal investigator (LFE) meets every 2 weeks with the researcher (SB) and research coordinator (BKE) to monitor trial conduct, i.e. closely reviewing the recruitment progress, data completeness and quality, contractual issues with the individual sites, training of counsellors and outcome assessors and budget administration. The senior researcher and assistant professor at the University of Copenhagen (JMK) make an annual external assessment of the study progress.

### Recruitment, consent and allocation to interventions

The enrolment period is planned to last 24 months: September 2018 to August 2020. Staff at each site screen individuals who meet the inclusion criteria and refer potential participants to the study. The researcher attends staff meetings to inform members about the project. Posters to encourage self-referral are displayed at the sites, e.g. in the reception areas in the community mental health centres. The researcher meets the candidates at the community mental health centre or in the candidate’s home. Oral and written information about the trial is provided. If the person decides to participate in the study, an informed consent form is signed. After baseline data are obtained, the participants are randomly assigned to either the control (standard mental healthcare) or experimental group (MA&R + standard mental healthcare) with a 1:1 allocation using the randomization module in REDCap (Research Electronic Data Capture) [44, 45]. The randomization sequence was generated by a researcher employed outside the research team and entered to RedCap. The randomization is stratified by sex. Varying block sizes, unknown to the research team, are used. To ensure concealment, the randomization schedule is stored away from the research team and the block sizes are not disclosed. The allocation is performed by the researcher, who informs the participants and the counsellors and who assigns participants to the interventions.

### Intervention

#### Meaningful Activities and Recovery

MA&R is a rehabilitation intervention tailored to persons with psychiatric disabilities, developed by the authors in collaboration with OTs and peer workers from community mental health centres. MA&R aims to enable and support meaningful activity engagement. The iterative process of testing and retesting the programme in loops with continuous feedback from service users, OTs and peer workers informed the programme delivery method, e.g. introducing one-to-one sessions and support as an alternative to home assignments, and presenting information in a manner sensitive to cognitive difficulties among participants. Additionally, the authors conducted a qualitative study that investigated how peer support and individualized support enhanced activity engagement in the everyday lives of persons with psychiatric disabilities participating in psychosocial interventions (to be submitted). Results from that study were integrated into the process of developing MA&R. Accordingly, MA&R is a multifaceted intervention where crucial elements to enable activity engagement include individualized support in the participant’s natural environment, peer workers and peer support in groups.

MA&R consists of 11 group sessions and 11 one-to-one sessions, which take place alternately. MA&R is delivered by a peer worker and an OT. In addition to the planned sessions, the participants are offered individualized support to engage in meaningful activities. The MA&R is a manualized intervention, with a workbook developed for the participants to support learning and personal exploration. MA&R utilized various methods such as didactic presentations, peer exchange, direct experiences with activities and personal exploration. The MA&R contains two courses: MA&R I and MA&R II, which complement each other. In MA&R I, the participants can explore and identify meaningful activities, while MA&R II supports anchoring new activities and habits in everyday life.

MA&R I consists of six group sessions and five one-to-one sessions and lasts for 7 weeks. Group sessions take place weekly and typically last 90 min. The group session is followed by a one-on-one session, which takes place one to 4 days after the group session.

MA&R II consists of five group sessions and six one-on-one sessions and lasts for approximately 5 months. In group sessions, participants are introduced to strategies for enabling activity engagement, e.g. making gradual changes by setting sub-goals. In addition to the planned sessions, the participants are offered individualized support in engaging in activities or initiating new activities.

Table 1 provides an overview of the themes and content in MA&R. In the community mental health centres, MA&R is delivered by the first author (STB), who is an OT, together with a peer worker. In the municipalities, MA&R is delivered by an OT or a social worker and a peer worker. The first author provides training and supervision of the OTs, social workers and peer workers in the municipalities. Fidelity will be monitored once per course with the MA&R Fidelity Scale, an instrument developed for the purpose. The evaluation will be done by external evaluators.

**Table 1:** Overview of themes, delivery mode (group-based or one-to-one sessions) and aims of the sessions in modules 1 and 2 in MA&R

Topic	Delivery mode^**1,2**^	Aim
***MA&R 1:*** *Overall aim: Learning about the role of meaningful activities in wellbeing and recovery and identifying meaningful activities in everyday life*		
**Introduction to MA&R I**	One-to-one session	▪ Learn about the structure and content in MA&R I ▪ Exchange mutual expectations of the course ▪ Talk about learning styles
**Flow**	Group session One-to-one session	▪ Emphasis on the main message in MA&R: what you do in daily life matters ▪ Learning about health and flow theory ▪ Identify activities associated with the experience of flow and/or wellbeing
**Activity patterns in daily life**	Group session One-to-one session	▪ Learning about activities and activity patterns ▪ Mapping own activities ▪ Identifying activities, routines, habits or contexts that influence well-being
**Recovery**	Group session One-to-one session	▪ Learning about recovery and how meaningful activities and day-to-day actions matter in the recovery process ▪ Learning about recovery and how it may unfold in daily life ▪ Abolish taboos and promote hope
**Telling personal stories through an activity perspective**	Group session One-to-one session	▪ Preparing narratives at the next group session ▪ Identify values, interests and personal resources through storytelling ▪ To tell one’s story in a safe forum, and to help each other explore personal resources and unique features
**Photo-voice: picturing the future**	Group session One-to-one session	▪ Consolidating MA&R I sessions by showing photos of meaningful activities in daily life
***MA&R 2:*** *Overall aim: To anchor meaningful activities, habits and routines in daily life*		
**Introduction to MA&R II**	One-to-one session	▪ Learn about the structure and content in MA&R II ▪ Exchange of mutual expectations of the course
**The PEO model (person-environment-occupation)** **Mapping opportunities for activity engagement**	Group session One-to-one session	▪ Learning about a problem-solving method to identify opportunities and barriers for meaningful activities ▪ Identifying strengths, opportunities and resources that can enable activity engagement by applying the PEO model
**One step at a time**	Group session One-to-one session	▪ Learning about habit changing and goal setting, e.g. by setting sub-goals ▪ Discussion about setting sub-goals, e.g. by trying to apply the goal staircase
**More energy in daily life**	Group session One-to-one session	▪ Learning strategies to balance activities and rest ▪ Planning and prioritizing energy-consuming activities ▪ Discussing and tailoring strategies to daily life
**Better overview of daily life**	Group session One-to-one session	▪ Learning about cognitive difficulties and personal strategies to tackle them ▪ Discussion and tailoring strategies to daily life
**Photovoice: meaningful activities now and in the future**	Group session One-to-one session (optional)	▪ Take photos of meaningful activities in relation to recovery or activity engagement ▪ Consolidating and evaluating MA&R II by showing photos of meaningful activities taken by the participants. Dialogue about maintain activity engagement during difficult periods in life
**Catching up**	One-to-one session	▪ Boost strategies and/or motivation for activity engagement, and clarify need for support

^1^In the sessions, a variety of methods are utilized: didactic presentations, dialogue, peer exchange, storytelling, worksheets and photovoice

^2^In addition to the planned sessions, participants are offered individualized support to initiate or sustain activity engagement. Examples of support are practical help, companionship when trying out new activities, looking for new opportunities for activities in the community, etc.

#### Standard mental healthcare

Standard mental healthcare in the CMCHs includes appointments with a psychiatrist, psychologist and/or care manager, in addition to psychoeducation, social skills training, peer-run recovery groups and relational support. Standard mental healthcare in the municipalities includes supported accommodation, drop-in centres, social activities, creative activities, self-esteem groups, peer services, skills-training, individualized support in activities of daily living (ADL) and relational support. Concomitant care, such as additional psychological counselling and National Acupuncture Detoxification Association (NADA), is permitted during the trial.

#### Modification and adherence

The intervention can be discontinued if the participants withdraw consent, or the investigators identify harms caused by the intervention, e.g. if the participants report mental health deterioration or symptom worsening due to participation. Adherence to the intervention is monitored and improved by allowing different parts to be delivered in a flexible manner, e.g. scheduling the one-to-one sessions according to the participant’s preferences. To enhance attendance, an SMS is sent as a reminder the same day as group sessions are held.

### Outcome measures

This study aims to investigate the effectiveness of MA&R on activity engagement and associated health outcomes, functioning, personal recovery and quality of life. The primary and secondary outcomes will be assessed twice, before randomization (baseline) and after intervention (follow-up, *t*_1_). Sociodemographic data and clinical measures are obtained at baseline, and harm measures are obtained at follow-up. Assessment methods are self-report questionnaires. Functioning will be rated by an assessor at baseline and register data obtained to identify harms.

#### Baseline measures

##### Sociodemographic data and clinical measures

Enrolled participants are asked to report their age, personal identification number (CPR number), housing status, educational level, marital status, number of children, income status, and information about psychiatric diagnoses and alcohol or substance abuse. Disability is assessed by the researcher using Mini-ICF Rating for Limitations of Activities and Participation in Psychological Disorders (Mini-ICF-APP), an observer rating instrument.

The MINI-ICF-App has been found to have substantial internal consistency (Cronbach *α* = 0.869–0.912), good test-retest (ICC 0.832) and interrater (ICC 0.886) reliability [43]. The MINI-ICF-App assesses capacity limitation within 13 domains, e.g. self-care, structuring tasks and relationships, and gives a total score within the range of 0 and 52, where a higher value indicates a more severe degree of disability.

#### Primary outcome

Changes in activity engagement between baseline and *t*_1_ are measured using the self-report version of the Profiles of Occupational Engagement in people with Severe Mental Illness (POES-S) [39], an instrument developed to assess time-use patterns of activity performance and the extent to which these patterns are characterized by engagement. POES-S consists of two parts: a 24- h, yesterday time-use diary sheet and a questionnaire with nine items reflecting activity engagement, e.g. daily rhythm of activity and rest, variety of activities POES-S gives a total score between 9 and 36, a higher score indicates greater activity engagement. POES-S has high internal consistency, *α* = 0.85 [21, 38, 39]. Changes in activity engagement will be assessed by comparison of the POES-S mean score with standard deviation (SD) and median at post-intervention (*t*_1_) between the experimental and control groups.

#### Secondary outcomes

Changes in functioning between baseline and *t*_1_ are measured with WHODAS 2.0 12-item version, a self-administered instrument consisting of 12 items derived from the WHODAS 2.0 36-item version [42, 46]. Disabilities within 12 domains of functioning, e.g. household responsibilities and community activities, are summarized to a total score between 12 and 60, where higher values indicate higher levels of disability. Scores on WHODAS 2.0 12-item version account for 85% of the variance in scores on the full 36-item version. The psychometric properties of WHODAS 2.0 12-item version are good with high internal consistency and excellent convergent validity [47, 48]. We also use the six subscales in the WHODAS 2.0 36-item version as an exploratory outcome measure. Changes in functioning will be assessed by comparison of WHODAS 2.0 12-item mean score with SD and median at post-intervention (*t*_1_) between the experimental and control groups. In addition, a mean score will be calculated for each of WHODAS 2.0 six subscales and compared between the experimental and the control groups at post-intervention (*t*_1_). Changes in personal recovery between baseline and *t*_1_ are measured with the Questionnaire about Process of Recovery (QPR) [40]. QPR measures the aspects of the personal recovery process, e.g. sense of agency and hope. In this study, we use the QPR 15-item version, which gives a total score between 0 and 70; higher scores are indicative of recovery. The QPR 15-item version has high internal consistency, test-retest reliability and convergent validity [40]. Changes in personal recovery will be assessed by comparison of QPR mean scores with SD and median at post-intervention (*t*_1_) between the experimental and control groups. Changes in quality of life between baseline and *t*_1_ are measured by the Manchester Short Assessment of Quality of Life (MANSA) [41]. MANSA contains 16 questions related to quality of life, e.g. satisfaction with life, job and financial situation. The sum score is 12–84, higher values indicate a greater quality of life. MANSA has shown reasonable psychometric properties in respect to concurrent validity, construct validity and internal consistency [41]. Changes in quality of life will be assessed by comparison of MANSA mean score with SD and median at post-intervention (*t*1) between the experimental and control groups. The Clinical Global Impression – Severity of Illness Scale (CGI-S) is used to measure the severity of mental illness. In this study, we use a self-reported version of CGI-S where the participants are asked to rate the severity of their current mental symptoms on a 7-point Likert scale from 1 (equal to no signs of mental illness) to 7 (the worst the disease has ever been). Studies show that CGI-S self-reported scores correlate with clinician-assessed scores (*r* = 0.34) [49]. Changes in severity of mental illness are assessed by comparison of CGI-S score mean, with SD and median at post-intervention (*t*_1_) between the experimental group and control group. Changes in health-related quality of life are measured with EuroQol (EQ-5D), a validated instrument that covers five dimensions: mobility, self-care, usual activities, depression/anxiety and pain, and includes a VAS scale of 0–100, with a higher score indicating a better health status. The EQ-5D can be transformed into an index score [50]. Changes in health-related quality of life will be assessed by comparison of EQ-5D mean scores with SD and median at post-intervention (*t*_1_) between the experimental group and control group.

### Blinding

Due to the nature of this trial’s intervention, the mental health professionals at the sites, the staff delivering MA&R and the participants cannot be blinded to the intervention allocation. The research assistant who collects follow-up data is blinded, and the participants are instructed not to disclose the allocation status to the research assistant. An employee outside the research team will extract data from REDCap on study completion to two separate Excel sheets, and group allocation will be coded with A and B to ensure blinding of the researcher while analysing data, drawing conclusions and writing up reports.

#### Data collection methods and management

The first author (STB) collects all data at baseline and is trained in using the MINI-ICF-app Social functioning scale through training videos provided by Hogrefe (https://www.hogrefe.co.uk/shop/mini-icf-app-social-functioning-scale.html). The participants can respond to the questionnaire in one of three ways: the questionnaire can be sent as an e-mail containing a link to answer the questions online; the participants can complete the questionnaire in a pen-and-paper format, or the participant can respond to the questionnaires with assistance from the researcher. How the participants respond to the questionnaire is listed in REDCap. Attendance at MA&R sessions is registered in the intervention-arm by the MA&R mentors facilitating the course. Follow-up data are obtained using the same questionnaires as used at baseline. Questionnaires are sent to all participants, including those who have decided to withdraw from the intervention, unless they have withdrawn their consent to participate in the study. The participants are reminded or prompted to complete the questionnaires through personal contact (SMS or phone call). To enhance adherence to the trial, participants are assisted if they wish by a research assistant at follow-up and the follow-up schedule is as flexible as possible to minimize the burden on the participants. Study data are collected and stored using REDCap, an electronic data capture tool hosted at the Capital Region of Denmark. REDCap is a secure, web-based software platform designed to support data capture for research studies [44, 45]. Access to data requires a two-step unique log-in system and is granted to researchers and staff affiliated with the project. A trial audit will not be conducted due to lack of funding. All physical data material (signed consent forms, case report forms and questionnaires) is stored in locked facilities. Table 2 shows an overview of the data collection.

**Table 2:** Schedule of data collection during the MA&R study period

Time point	Enrolment	Baseline	Randomization	Intervention period	Eight months follow-up
Eligibility screening	x				
Informed consent	x				
Allocation			x		
Register sheets for attendance in MA&R					
Self-assessment data					
- POES-S		x			x
- WHODAS 2.0		x			x
- QPR		x			x
- MANSA		x			x
- Euro-QoL		x			x
- CGI-S		x			x
CRF including MINI ICF Social Functioning Scale		x			
Registry data					x

*CRF* case report form

Data quality will be promoted through a variety of procedures. First, monthly data monitoring is conducted by the research coordinator (BKE) for data completeness and verification of data, e.g. personal identification number. Missing or invalid data are reported directly to the researcher (SB). Secondly, REDCap has functions designed to detect missing data and certain errors in data, e.g. invalid values. Thirdly, a subset of case report forms and questionnaires will be selected by a researcher outside the research team to check accurate data entry prior to analysis. Lastly, final data control will be performed before analysis by the research coordinator. Participant files are stored for up to 10 years after study completion.

### Sample size

The sample size is calculated based on the primary hypothesis to detect a minimal but clinically significant difference between the intervention group and the control group on POES-S (increased engagement in meaningful activities). In a recent RCT testing, the intervention Balancing Everyday Life (BEL) in a similar population as that in this study, Eklund et al. found a small effect of 1.4 points measured on POES-S (*d* = 0.27) [21]. As MA&R has a more comprehensive format (in terms of duration and costs) than BEL, the clinically significant difference between the study groups is set to 3 points, corresponding to a moderate effect size (Cohens *d* = 0.5). Based on the BEL trial, we assume the standard deviation in the study population to be 6. To achieve a statistical power of 80% and a significance level of 5%, a total of 128 participants must be included in this study; 64 in each group to detect this difference.

### Statistical methods

The main outcome measure is activity engagement, measured by POES-S. To test the research hypothesis, the differences between the intervention group and the control group will be analysed using ANOVA to determine the statistical significance. Effect sizes to judge clinical relevance will be calculated by Cohen’s *d*.

In accordance with the intention-to-treat principles, “multiple multivariate imputations” will be used and all co-variates of supposed prognostic significance will be used to impute a distribution of missing data.

The continuous power measurements will be analysed with a repeated measurement model in mixed model analyses with unstructured variance. The prerequisite for using this analysis and for the use of multiple imputations is that data are missing at random or data missing completely at random as opposed to non-ignorable non-response. This distinction is important as repeated measurements and multiple imputations are both models based on a statistical estimation of non-existent responses, and the prerequisites for this estimation must be met for the analyses to be valid.

Whether the prerequisites for using repeated measurement and multiple imputations are present will be analysed by performing a drop-out analysis. Significant prognostic characteristics of the non-follow-up participants will be compared with those of the participants remaining in the trial. Variables where there is a difference between participants and non-participants will be included as co-variables in the analyses. Data analyses will be based on the intention-to-treat principle. Data from all participants will be included corresponding to the group to which they have been allocated. As a supplementary analysis, we will carry out per-protocol where complete cases will be analysed.

Differences between the intervention group and the control group regarding secondary outcomes will be analysed applying the same statistical methods because all outcome variables are continuous.

A detailed statistical analysis plan will be prepared before initiating analysis and uploaded at clinicaltrials.gov

### Monitoring

Based on trials with similar interventions, such as BEL, we do not anticipate any adverse risks or reactions to MA&R. Harms will be identified as unfavourable changes in psychiatric symptoms, hospitalizations (somatic and psychiatric) and deaths (all causes) and will be evaluated at follow-up. Data on psychiatric symptoms will be obtained through questionnaires (GCI-S), while data on hospitalizations and deaths will be obtained from The Danish National Patient Registry and The Cause of Death Register. Adverse events will be reported in trial publications, to the sites, sponsors and the ethics committees. Unexpected harms are collected during the study period, through e-mail, telephone or face-to-face communication with participants in the intervention arm and counsellors. Examples of unanticipated adverse events attributed to the intervention and patient population can be elevated levels of stress, e.g. irritability or self-blame, and will be reported to the researcher by the participant or mental health staff. Harms are discussed in the research group at the bi-weekly meetings. Unexpected adverse events will be reported to the sites and ethics committees and in trial publications.

### Dissemination policy

The results will be published in at least one article in a peer-reviewed scientific journal. The results will also be published in a PhD dissertation and in professional journals, after scientific publication. Positive, negative and inclusive results will be published. Results from the research project will also be presented at scientific conferences. Authorship is determined by the Vancouver criteria (http://www.icmje.org).

## Discussion

Psychiatric disabilities are prevalent across a range of mental health disorders and can entail tremendous costs for both the individual and society [3–5, 51–53]. Persons with mental illness may experience daily life as a vicious circle of lack of energy, inertia and isolation [9, 10]. Engagement in activities perceived as meaningful can play a crucial part in the recovery process [24, 26, 34]. However, evidence is scarce of how to support persons with psychiatric disabilities in re-engaging and sustaining meaningful activity engagement. This necessitates interventions that directly target activity engagement. This study aims to investigate the effectiveness of a novel rehabilitation intervention designed to enable engagement in activities that are meaningful in the participants’ lives. Evidence-based interventions targeting activity engagement are few, and this trial will contribute with new knowledge about rehabilitation interventions among individuals with psychiatric disabilities. The CONSORT 2010 statement [37] and the SPIRIT 2013 guidelines [35, 36] informed the study design, and the trial has a sample size sufficiently large to detect a moderate effect size of MA&R if it exists. This is a multicentre study, with sites in community mental health centres and municipalities, which may increase the potential for implementation in various mental health settings, if the findings are positive. The pragmatic design of the trial, regarding in- and exclusion criteria and settings, enables assessment of the intervention’s effect under real-life conditions. Patients and clinicians were involved in designing, pilot testing and giving the feedback that informed the development of MA&R. Research questions and outcome measures were discussed in the research group consisting of clinicians and researchers, with input from mental health professionals. The rationale for choosing the outcome measure is due to its major clinical relevance. The burden of participating in the research was carefully assessed by pilot testing the questionnaires among lay members, which narrowed down the number of outcome measures.

Nevertheless, this study has methodological weaknesses. First, it is not possible to blind the participants and the professionals to allocation. The researcher who developed MA&R is involved in delivering the interventions in the community mental health centres. This increases the risk of performance and expectancy bias and will be considered when discussing the results of the trial. Second, it has not been possible to stratify pr. site, but this potential confounding factor will be considered during stages of the statistical analyses. Third, MA&R is delivered as an add-on to standard mental healthcare treatment/service. Participants are encouraged to continue to use the standard mental health services, but no data exist on the quantity of participants’ mental health service use.

Moreover, the focus of the trial is on the effect of the intervention but does not incorporate participants’ perspectives on the impact of the intervention. Therefore, a qualitative study of MA&R will be conducted if additional funding is generated. We plan to conduct health economic analysis, using data from EQ-5D, if further funding is obtained.

### Trial status

The trial was launched in September 2018 and is currently in the recruitment phase. Recruitment is anticipated to be completed by 30 August 2020. The data collection period is planned to end in February 2021. This is protocol version No 4, from 19 November 2019.

## Supplementary information

**Additional file 1:.** Data category and information.

## Data Availability

It is not possible to obtain access to the final trial dataset as data sharing is not permitted by Danish law.

## Abbreviations

BEL: Balancing Everyday Life
CRF: Case rapport form
CONSORT: Consolidated Standards of Reporting Trials
CORE: Copenhagen Research Center for Mental Health
EQ-5D: EuroQol
GDPR: General Data Protection Regulation
ICF: International Classification of Functioning, Disability and Health
MANSA: Manchester Short Assessment of Quality of Life
MA&R: Meaningful Activities and Recovery
MINI-ICF-App: Mini-ICF Rating for Limitations of Activities and Participation in Psychological Disorders
Self-rapport version – POES-S: Profiles of Occupational Engagement in people with Severe Mental Illness
QPR: Questionnaire about Process of Recovery
SPIRIT: Standard Protocol Items: Recommendations for Interventional Trials
CGI-S: Clinical Global Impression – Severity of Illness Scale
WHODAS 2.0 12: WHO Disability Assessment Schedule 2.0
WHO: World Health Organization
