# Supplementary material for: Meaningful Activities and Recovery (MA&R): the effect of a novel rehabilitation intervention among persons with psychiatric disabilities on activity engagement—study protocol for a randomized controlled trial
Source: Trials. 2020 Sep 14;21:789. doi: 10.1186/s13063-020-04722-3 (PMC7491082; doi:10.1186/s13063-020-04722-3)
Supplement: Supplementary file 1 — Additional file 1:. Data category and information. [file 13063_2020_4722_MOESM1_ESM.docx]

| **Data category** | **Information** |
| --- | --- |
| Primary registry and trial identifying number | ClinicalTrials.gov  NCT 03963245 |
| Date of registration in primary registry | 15 th May 2019 |
| Secondary identifying numbers | N/A |
| Source(s) of monetary or material support | Trygfoundation, The Danish Occupational Therapy Association`s Researchs Foundation and Mental Health Center in Capital Region of Denmark |
| Primary sponsor | Ph.d MD Lene Falgaard Eplov  Copenhagen Research Center for Mental Health (CORE) |
| Secondary sponsor(s) |  |
| Contact for public queries | *Phd. Student Siv Therese Bogevik Bjørkedal, Copenhagen Research Center for Mental Health (CORE)*  Phone: 00 45 20594538 |
| Contact for scientific queries | Phd. Student Siv Therese Bogevik Bjørkedal, Copenhagen Research Center for Mental Health (CORE) |
| Public title | *Meaningful Activities and Recovery (MA&R). The effect of a novel rehabilitation intervention to people with psychiatric disabilities on activity engagement: Study protocol for a randomized controlled trial.* |
| Scientific title | *Meaningful Activities and Recovery (MA&R). The effect of a novel rehabilitation intervention to people with psychiatric disabilities on activity engagement: Study protocol for a randomized controlled trial.* |
| Countries of recruitment | Denmark |
| Health condition(s) or problem(s) studied | Recovery-oriented rehabilitation, Psychiatric disabilities |
| Intervention(s) | Active comparator: Meaningful Activities and Recovery (MA&R) a recovery-oriented rehabilitation intervention in addition to standard mental health care Standard care: Standard mental health care |
| Key inclusion and exclusion criteria | Ages eligible for study: ≥18 years Sexes eligible for study: both Accepts healthy volunteers: no Inclusion criteria: adult individuals (≥ 18 years), service users in community mental health centers and/or mental health services in three Danish municipalities (Copenhagen, Odense and Svendborg). Diagnosed with mental disorder and having psychiatric disabilities, assessed by MINI-ICF-App Social Functioning Scale, at baseline.  Exclusion criteria: Diagnosed with dementia, forensic status, need for translator (do not understand Danish) and substance or alcohol abuse that interfere with attending group sessions. |
| Study type | Interventional Allocation: randomized Intervention model: parallel assignment Masking: single blind (outcomes assessor) Primary purpose: Rehabilitation |
| Date of first enrolment | September 2018 |
| Target sample size | 128 |
| Recruitment status | Recruiting |
| Primary outcome(s) | Activity engagement (self-reported) |
| Key secondary outcomes | Functioning, Personal Recovery, Quality of Life |
